# Fecal Microbiota Transplantation Increases Colonic IL-25 and Dampens Tissue Inflammation in Patients with Recurrent Clostridioides difficile

**DOI:** 10.1128/mSphere.00669-21

**Published:** 2021-10-27

**Authors:** N. Jan, R. A. Hays, D. N. Oakland, P. Kumar, G. Ramakrishnan, B. W. Behm, W. A. Petri, C. Marie

**Affiliations:** a Department of Medicine, Infectious Diseases and International Health, University of Virginia School of Medicine, Charlottesville, Virginia, USA; b Department of Medicine, Gastroenterology & Hepatology, University of Virginia School of Medicine, Charlottesville, Virginia, USA; c Department of Pathology, University of Virginia School of Medicine, Charlottesville, Virginia, USA; d Department of Microbiology, Immunology and Cancer Biology, University of Virginia School of Medicine, Charlottesville, Virginia, USA; e Department of Biochemistry and Molecular Genetics, University of Virginia School of Medicine, Charlottesville, Virginia, USA; University of Iowa

**Keywords:** *C. difficile* infection, fecal microbiota transplantation, type 2 immunity

## Abstract

Clostridioides difficile infection (CDI) is the most common hospital-acquired infection in the United States. Antibiotic-induced dysbiosis is the primary cause of susceptibility, and fecal microbiota transplantation (FMT) has emerged as an effective therapy for recurrence. We previously demonstrated in the mouse model of CDI that antibiotic-induced dysbiosis reduced colonic expression of interleukin 25 (IL-25) and that FMT protected in part by restoring IL-25 signaling. Here, we conducted a prospective study in humans to test if FMT induced IL-25 expression in the colons of patients with recurrent CDI (rCDI). Colonic biopsy specimens and blood were collected at the time of FMT and 60 days later. Colon biopsy specimens were analyzed for IL-25 protein levels, total tissue transcriptome, and epithelium-associated microbiota before and after FMT, and peripheral immune cells were immunophenotyped. FMT increased alpha diversity of the colonic microbiota and levels of IL-25 in colonic tissue. In addition, FMT increased expression of homeostatic genes and repressed inflammatory genes. Finally, circulating Th17 cells were decreased post-FMT. The increase in levels of the cytokine IL-25 accompanied by decreased inflammation is consistent with FMT acting in part to protect from recurrent CDI via restoration of commensal activation of type 2 immunity.

**IMPORTANCE** Fecal microbiota transplantation (FMT) is an effective treatment for C. difficile infection for most patients; however, introducing a complex mixture of microbes also has had unintended consequences for some patients. Attempts to create a standardized probiotic therapeutic that recapitulates the efficacy of FMT have been unsuccessful to date. We sought to understand what immune markers are changed in patients undergoing FMT to treat recurrent C. difficile infection and identified an immune signaling molecule, IL-25, that was restored by FMT. This finding indicates that adjunctive therapy with IL-25 could be useful in treating C. difficile infection.

## INTRODUCTION

Clostridioides difficile is an opportunistic pathogen that can cause life-threatening diarrhea and colitis. Initial C. difficile infection (CDI) is typically treated with antibiotics such as vancomycin or fidaxomicin (1). The efficacy of standard antibiotic therapy for primary CDI is 58 to 78% and decreases substantially after the first recurrence of disease (2). Approximately 20 to 30% of patients develop recurrent CDI (rCDI) within 2 weeks of completion of therapy (3). Fecal microbiota transplant (FMT) is an effective treatment for rCDI: a recent meta-analysis found the overall efficacy of FMT to be 76.1%, with lower efficacy for treating patients with refractory CDI as opposed to rCDI (63.9% versus 79%) (4).

Despite the increasing use of FMT for treatment of rCDI, the mechanisms of action of FMT are poorly defined. It is hypothesized that FMT has direct inhibitory effects on C. difficile via niche exclusion, nutrient competition, and the production of antimicrobial peptides (5–7). FMT may also induce changes in the host intestinal epithelium that increase resistance to recurrence of disease via fortification of the mucus layer and differentiation and proliferation of intestinal epithelial cells (8).

One reason the mechanisms of FMT are unclear is that host immune responses can vary greatly and have complex effects on C. difficile infection severity, as well as the efficacy of FMT (9–12). Type 1 responses via type 1 innate lymphoid cells (ILC1s) have been shown to be protective (13), type 17 immune responses have been related to increased host damage, and type 2 immune responses via ILC2s have been related to tissue repair via eosinophil recruitment (14, 15). It is therefore important to consider patient variability and the interplay between microbiota and host immune response when analyzing the mechanisms behind FMT as a treatment for rCDI.

We hypothesized that FMT acts to protect from rCDI in part by restoring commensal-bacterium signaling to the innate immune system via the intestinal epithelial produced cytokine IL-25. To test this hypothesis, subjects with rCDI undergoing FMT were prospectively enrolled in a clinical study, and colonic biopsy specimens and peripheral blood were collected pre- and post-FMT. The goal was to analyze the local and peripheral immune responses induced by FMT. Colonic biopsy specimens were used to assess the transcriptional response of the intestinal epithelium to FMT by RNA sequencing and the protein-level response using immunoassays for cytokines. Intestinal and peripheral immune cell populations were analyzed by high-dimensional flow cytometry. In addition, host epithelium-associated bacteria were identified by 16S rRNA gene sequencing from the colonic biopsy specimens.

## RESULTS

### Participants.

We recruited 10 patients undergoing FMT to treat rCDI infections; of these, 90% had at least 2 recurrences of rCDI before FMT. Of the 10 participants recruited for the study, 6 completed both visits, while 4 did not return for the 60-day follow-up visit after FMT. All patients had zero CDI recurrences and zero hospitalizations in the 60-day follow-up period after FMT. Additionally, FMT prevented further CDI recurrences for at least 2 years. However, though every patient was clinically cured with an absence of diarrhea and negative CDI test, of the 6 that completed both visits, two patients had persistent symptoms consistent with postinfectious irritable bowel syndrome (IBS) as defined by Rome IV criteria (16). The 6 participants that completed the second visit were female. The clinical characteristics are summarized in Table 1.

**TABLE 1:** Demographics and baseline clinical data of patients that underwent FMT treatment for recurrent C. difficile infection

Clinical characteristic	Value for cohort	
	Total (*n* = 10)	With second visit (*n* = 6)
% (no./total) with second visit	60 (6/10)	100 (6/6)
Mean (SD) age at FMT, yrs	71 (7.71)^*a*^	69.83 (8.70)
% (no./total) women	90 (9/10)	100 (6/6)
Mean (SD) BMI at FMT	31.76 (8.91)^*a*^	28.79 (8.68)
Mean no. (SD) of CDI recurrences per patient pre-FMT	3.1 (0.60)	3.2 (0.75)
% (no./total) with no. of recurrences		
2	10 (1/10)	17 (1/6)
3	70 (7/10)	50 (3/6)
4	20 (2/10)	33 (2/6)
% (no./total) treated with vancomycin prior to FMT	100 (10/10)	100 (6/6)
% (no./total) who developed IBS-like symptoms post-FMT	20 (2/10)	33.3 (2/6)
% (no./total) with CDI recurrences post-FMT	0 (0/10)	0 (0/6)
% (no./total) with same FMT donor as another subject	40 (4/10)	50 (3/6)
% (no./total) who did not have same FMT donor as another subject	60 (6/10)	50 (3/6)

a Missing data for one patient.

### FMT increased colonic type 2 cytokines.

To evaluate changes in immune cell signaling in response to FMT, we measured 47 cell signaling proteins in colonic biopsy tissue lysates (see Table S1 in the supplemental material). Interleukin 25 (IL-25) and IL-4 cytokines were of particular interest, since they are related to eosinophil recruitment and a type 2 immune response, which we predicted *a priori* to be restored by FMT (15). In addition, because inflammasome activation has been shown to be central to C. difficile infection pathogenesis and IL-1 was specifically identified in a previous study (17), IL-1a and IL-1b were also selected *a priori* for analysis in the trial outcomes.

10.1128/mSphere.00669-21.5 TABLE S1 Pre- versus post-FMT protein concentrations from Luminex assays (linear mixed controlled by patient with Benjamini-Hochberg adjustment). Download Table S1, DOC file, 0.08 MB. Copyright © 2021 Jan et al. 2021 Jan et al. https://creativecommons.org/licenses/by/4.0/ This content is distributed under the terms of the Creative Commons Attribution 4.0 International license.

**FIG 1:**
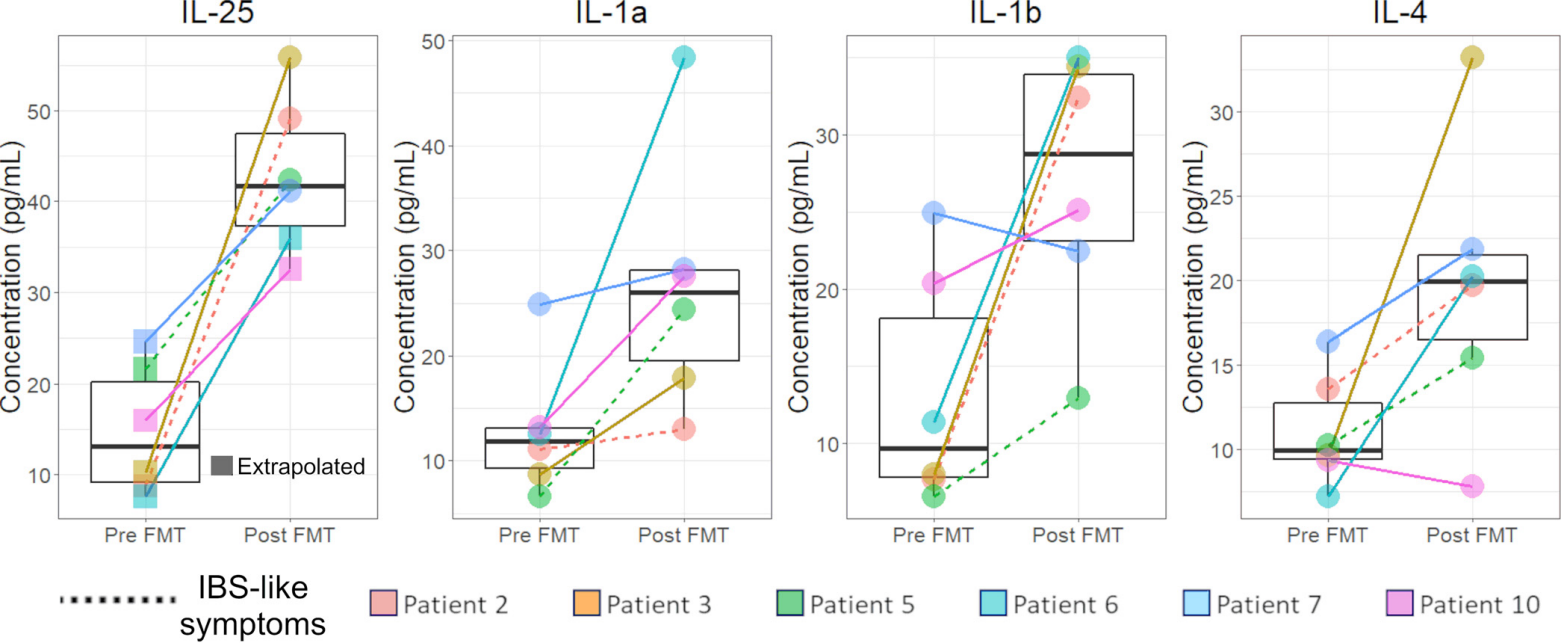
FMT increased Th2 cytokine levels in the colon. Levels of tissue cytokines were quantified by Luminex assay. The median values and interquartile ranges (IQR) of tissue cytokines before and after FMT are shown. A linear mixed effect model with patient as a random variable was the statistical test used to calculate *P* values. There were no significant differences in total protein concentration between pre- and post-FMT in the samples used for the Luminex assays (data not shown). Normalizing by the total protein concentration in each sample yielded similar results (Fig. S1). MFIs outside the standard curve range were extrapolated (squares).

We found that protein concentrations of IL-1a, IL-1b, and IL-25 were significantly increased after FMT (linear mixed effect model, *P* < 0.05) with a trend toward increased IL-4 concentrations after FMT (*P* = 0.058) (Fig. 1). Taking out the two patients that developed IBS-like symptoms, we found that the increase in IL-1a and IL-1b were no longer significant (*P* > 0.1 and *P* > 0.05, respectively), and IL-4 increase was still not significant (*P* > 0.1), while the increase in IL-25 was still significant (*P* < 0.05), suggesting that IBS-like symptoms may influence the concentrations of specific cytokines.

10.1128/mSphere.00669-21.1 FIG S1 Normalizing by the total protein concentration, IL-25 was still shown to increase following FMT. Of the four cytokines of interest, only IL-25 was significantly increased after FMT (linear mixed effect model, *P* < 0.01). Taking out the two patients that developed IBS-like symptoms post-FMT, we found that this increase was still significant (*P* < 0.05). Download FIG S1, TIF file, 0.5 MB. Copyright © 2021 Jan et al. 2021 Jan et al. https://creativecommons.org/licenses/by/4.0/ This content is distributed under the terms of the Creative Commons Attribution 4.0 International license.

In the parallel transcriptomic analysis, while IL-25 transcripts could not be identified, FMT modestly increased the expression of *IL1a* (log_2_ fold change [log_2_FC] = 0.83) and *IL1b* (log_2_FC = 0.47), though these increases were not significant (Fig. S2A and B) and none of these cytokine genes was identified as a differentially expressed gene. There was an increase in the transcript levels of the IL-1 inhibitor IL1RA (encoded by *IL1RN*; log_2_FC = 0.83) following FMT (Fig. S2C). *IL1A* transcripts were negatively correlated with IL-1a protein levels (*r* < −0.6), while *IL1B* transcripts were positively correlated with IL-1b protein levels (*r* > 0.6) (Fig. S2D). *IL1RN* transcripts were positively correlated with IL1RA protein concentration (*r* > 0.7) (Fig. S2D).

10.1128/mSphere.00669-21.2 FIG S2 Correlations between different analyses show some relationships between gene transcription, protein concentration, and epithelial microbial abundance. There was moderate induction of *IL1A* (A), *IL1B* (B), and *IL1RN* (C) transcription from pre- to post-FMT, and these increases were not significant (*P* > 0.1, Wald test). For all three genes, the transcriptional response to FMT was variable from patient to patient. For the transcription of *IL1A*, some patients exhibited little change (patient 3) or a decrease from pre- to post-FMT (patient 6). For the transcription of *IL1B*, patient 10 exhibited a decrease in transcription, and there was little change in transcription for patients 5 and 7. For the transcription of *IL1RN*, patients 5 and 10 showed decreases in transcription. *IL1A* gene transcription had a negative correlation with IL-1a protein concentration, but for genes *IL1B* and *IL1RN*, there were positive correlations with their respective proteins. The genus *Akkermansia* had a positive correlation with bile secretion pathway genes (E) and a negative relationship with IL-25 protein concentration (F). Download FIG S2, TIF file, 0.5 MB. Copyright © 2021 Jan et al. 2021 Jan et al. https://creativecommons.org/licenses/by/4.0/ This content is distributed under the terms of the Creative Commons Attribution 4.0 International license.

### Transcriptional responses to FMT.

**FIG 2:**
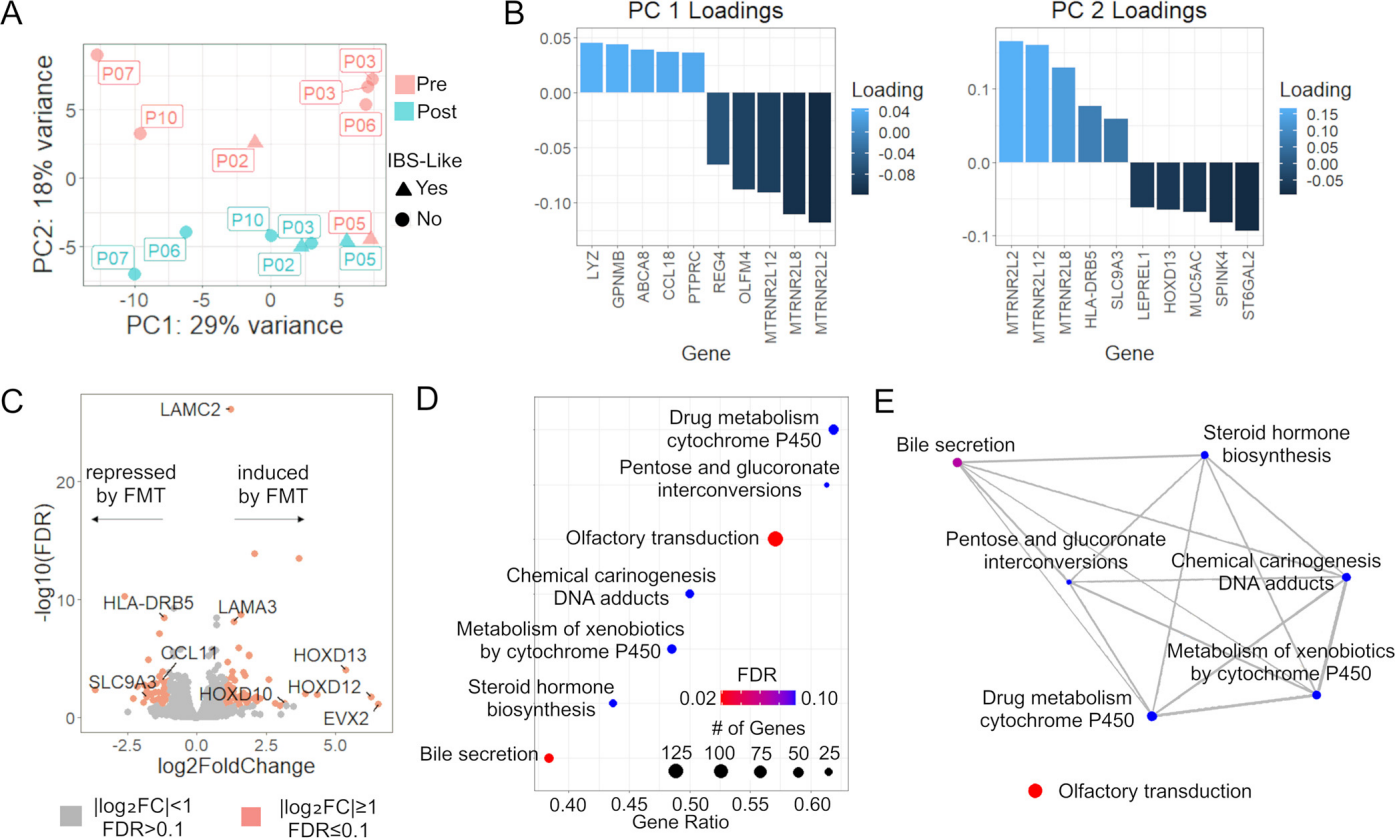
FMT driven variation in the colonic transcriptome. (A) Principal-component analysis of gene expression data identifies separation between pre-FMT (red) and post-FMT (green) samples. Batch effects were minimal, as noted by how closely pre-FMT patient 3 (P03) samples from 2 different batches grouped. PC1 captured some variance induced by the development of IBS-like symptoms post-FMT in patients 2 (P02) and 5 (P05) clustered on the negative side of PC1. PC2 captured the variance related to FMT, where each patient consistently had a higher PC2 value before FMT and a lower PC2 value after FMT. (B) PC1 loadings were driven by *OLFM4* (positive) and *LYZ* (negative), while PC2 loadings were driven by *HLA-DRB5* (positive) and *ST6GAL2* (negative). (C) Volcano plot of differentially expressed genes after FMT. Orange indicates genes that met the threshold of a log_2_FC value of ≥1 and an FDR of ≤0.1 (Wald test, Benjamini-Hochberg adjustment). Genes mentioned in the text are labeled here, including homeobox genes, laminins, and some genes related to immune responses. (D) Overrepresented pathways from pre- to post-FMT related to bile acids had overlapping genes. Using the FCs from DEGs from pre- to post-FMT, overrepresented pathways were found using KEGG gene lists (enrichment score test, Benjamini-Hochberg adjustment). (E) Overlaps in pathways were mapped to find common themes.

To assess the transcriptional response in colonic tissue, we compared gene expression in colonic biopsy specimens obtained immediately prior to FMT to those obtained 60 days after FMT. Principal-component analysis (PCA) of the normalized gene counts from transcriptome sequencing (RNA-seq) was done to investigate unsupervised groupings by visit, patient, or development of IBS-like symptoms post-FMT (Fig. 2A). Principal component 2 (PC2) separated the samples into pre- versus post-FMT groups, while PC1 was related to patients that developed IBS-like symptoms after FMT. Negative loading unique to PC1 was driven by *OLFM4* (Fig. 2B). *OLFM4* encodes olfactomedin-4, a selective marker of inflammation in the colonic epithelium (18). The largest positive loading, *LYZ*, encodes lysozyme which has been found previously to be expressed at lower levels in IBS patients than in healthy controls (19). The top loadings uniquely driving PC2 were *ST6GAL2* (negative) and *HLA-DRB5* (positive). *ST6GAL2* encodes a sialyltransferase. Specifically in relation to the gastrointestinal system, sialic acid catabolism has been shown to be related to intestinal inflammation and microbial dysbiosis (20–22), though the specific role of ST6GAL2 has not yet been established. *HLA-DRB5* encodes an HLA class II protein which is specific to antigen presentation for immune cells and is related to ulcerative colitis (23). Three genes overlapped between PC1 and PC2 loadings, *MTRNR2L12*, *MTRNR2L8*, and *MTRNR2L2*, which encode isoforms of the mitochondrial peptide humanin, which has been shown to have cytoprotective effects (24).

We also performed hierarchical clustering of the 30 most variable genes before and after FMT (Fig. S3). The dendrogram branched first between a group without any patients that developed IBS-like symptoms post-FMT (Fig. S3, right) and a group that included the two patients that developed IBS-like symptoms (left). For the no-IBS-like-symptom group, the second level of hierarchy was the split between pre- and post-FMT samples, before splitting by patient. The group that contained samples from patients that developed IBS-like symptoms were then split by patient before being split into pre- versus post-FMT. The two patients that developed IBS-like symptoms post-FMT did not cluster together, suggesting that patient-specific transcriptional responses vary even among patients with similar IBS-like symptoms.

10.1128/mSphere.00669-21.3 FIG S3 Hierarchical clustering of the top 30 variable genes by standard deviation of RNA-seq counts show some separation between patients that developed IBS-like symptoms post-FMT and those that did not. Colored boxes near the top of the plot indicate the visit (purple for pre-FMT and green for post-FMT), patient ID, and whether the patient developed IBS-like symptoms post-FMT (orange for no IBS-like symptoms and pink for developing IBS-like symptoms), while below that is a heat map of the normalized counts scaled around zero, where lower counts are blue and higher counts are red. In the first level of hierarchical clustering, the samples were separated into a group consisting of 5 samples from patients that did not develop IBS-like symptoms post-FMT and another that included all samples from patients that did develop IBS-like symptoms post-FMT. Download FIG S3, TIF file, 0.7 MB. Copyright © 2021 Jan et al. 2021 Jan et al. https://creativecommons.org/licenses/by/4.0/ This content is distributed under the terms of the Creative Commons Attribution 4.0 International license.

### FMT induces transcriptional programs of tissue repair and suppresses inflammatory responses.

We analyzed differentially expressed genes (DEGs) between pre- and post-FMT using a grouped model comparing all patients before and after FMT. Based on the unsupervised analyses of PCA (Fig. 2A) and hierarchical clustering (Fig. S2), we incorporated patient ID into the model to account for patient-to-patient variability. A total of 103 DEGs were identified (log_2_FC ≥ 1; false discovery rate [FDR] ≤ 0.1), of which 62 were induced while 41 were repressed by FMT (Fig. 2C). Some notable induced genes include homeobox genes (*EVX2*, *HOXD10*, *HOXD12*, and *HOXD13*) and laminin genes (*LAMC2* and *LAMA3*) (Table 2). Homeobox genes have been shown to be related to development and differentiation of the intestinal epithelium (25, 26), while laminins are components of the basal lamina which have been shown to be absent in ulcerative colitis (27). Some notable repressed genes include *HLA-DRB5*, *CCL11*, and *SLC9A3* (Table 2). *HLA-DRB5* is a class II allele involved in antigen presentation to CD4 T cells and appeared in the above PCA as being a positive loading to a component separating pre- and post-FMT. *CCL11*, also known as the eotaxin-1 gene, is proinflammatory and involved in eosinophil chemotaxis (Fig. 2C) (28). *SLC9A3* is related to bile acid elimination and diarrhea (29).

**TABLE 2:** Differentially expressed genes between pre- and post-FMT sorted by increasing FDR (Wald test adjusted by the Benjamini-Hochberg method)

Gene	*P* value	FDR	Log_2_FC
Genes induced by FMT			
*EVX2*	2.00E−03	7.67E−02	6.55
*HOXD12*	2.38E−04	1.81E−02	6.28
*HOXD13*	1.80E−07	1.02E−04	5.37
*OR51E2*	1.16E−04	1.11E−02	4.35
*CPB1*	1.06E−04	1.04E−02	3.91
*ST6GAL2*	6.80E−18	3.28E−14	3.67
*HOXD10*	1.71E−03	6.95E−02	3.16
*PRSS22*	3.08E−03	9.95E−02	2.99
*MUC5AC*	1.72E−03	7.00E−02	2.81
*MMP3*	2.21E−06	6.53E−04	2.60
*TNNC1*	4.48E−04	2.75E−02	2.27
*INSL5*	8.42E−04	4.38E−02	2.17
*KIAA1549L*	2.62E−04	1.95E−02	2.13
*SERPINB5*	1.76E−18	1.28E−14	2.08
*SLC6A14*	4.57E−04	2.78E−02	2.05
*TRIM29*	7.80E−09	5.89E−06	1.89
*PAPPA2*	6.11E−09	4.92E−06	1.88
*SPINK4*	5.79E−04	3.30E−02	1.85
*PALM2*	1.20E−03	5.60E−02	1.81
*REG4*	1.66E−04	1.43E−02	1.79
*CALN1*	6.89E−04	3.72E−02	1.78
*CLGN*	6.73E−07	2.97E−04	1.74
*KIF5C*	3.22E−07	1.56E−04	1.70
*MMP1*	7.24E−05	8.13E−03	1.67
*LEPREL1*	7.38E−13	1.78E−09	1.59
*TFPI2*	1.87E−04	1.52E−02	1.56
*AGTR1*	5.01E−04	2.98E−02	1.56
*MYBPC1*	2.37E−03	8.46E−02	1.55
*ME1*	2.35E−04	1.81E−02	1.55
*TM4SF1*	9.97E−10	1.20E−06	1.52
*IRX2*	8.90E−04	4.56E−02	1.52
*S100P*	4.65E−04	2.81E−02	1.46
*TSLP*	1.68E−03	6.89E−02	1.45
*CCDC85A*	1.92E−03	7.49E−02	1.42
*LYPD6B*	3.94E−04	2.56E−02	1.41
*SST*	2.37E−04	1.81E−02	1.39
*PLLP*	2.96E−06	7.84E−04	1.37
*LAMA3*	4.63E−12	7.46E−09	1.33
*OSR1*	5.00E−05	6.41E−03	1.33
*FIBCD1*	2.96E−03	9.73E−02	1.31
*FAM135B*	1.98E−05	3.22E−03	1.30
*MME*	2.69E−07	1.39E−04	1.28
*SLC6A12*	1.34E−06	4.32E−04	1.27
*EREG*	1.28E−06	4.23E−04	1.25
*CTSL2*	7.56E−05	8.43E−03	1.24
*MLF1*	3.48E−05	4.80E−03	1.23
*LAMC2*	5.03E−31	7.29E−27	1.22
*CYP2W1*	2.40E−03	8.52E−02	1.20
*TNFRSF12A*	2.43E−05	3.67E−03	1.20
*CTSE*	9.68E−06	1.89E−03	1.17
*RBM24*	7.62E−04	4.05E−02	1.16
*GDF15*	1.69E−04	1.43E−02	1.15
*SULT1C2*	5.85E−05	7.11E−03	1.13
*SCEL*	1.90E−03	7.46E−02	1.12
*RHOD*	8.83E−06	1.83E−03	1.11
*BMP7*	3.44E−08	2.17E−05	1.10
*GCG*	1.92E−06	5.93E−04	1.06
*RFX6*	1.36E−03	6.03E−02	1.06
*COL2A1*	2.67E−03	9.20E−02	1.02
*SPINK5*	1.58E−05	2.69E−03	1.02
*TDRD6*	2.30E−03	8.28E−02	1.01
*SH3TC2*	1.78E−04	1.48E−02	1.00

Genes repressed by FMT			
*PRHOXNB*	3.36E−05	4.69E−03	−3.66
*HSD3B2*	1.53E−14	5.55E−11	−2.60
*COL6A5*	4.24E−04	2.70E−02	−2.27
*CLC*	1.62E−05	2.72E−03	−2.13
*KCNG1*	1.13E−03	5.43E−02	−1.92
*CREB3L3*	8.31E−06	1.77E−03	−1.88
*SLC9A3*	2.50E−04	1.90E−02	−1.81
*SH2D6*	4.73E−05	6.23E−03	−1.80
*PLA2G12B*	1.57E−05	2.69E−03	−1.80
*NR1H4*	1.08E−04	1.04E−02	−1.78
*VEPH1*	2.14E−08	1.41E−05	−1.75
*SH2D7*	9.90E−06	1.89E−03	−1.66
*POU2F3*	9.72E−05	9.93E−03	−1.64
*TTBK1*	6.68E−04	3.63E−02	−1.58
*C11orf53*	6.91E−04	3.72E−02	−1.57
*SLC6A19*	6.24E−05	7.29E−03	−1.50
*NETO2*	6.06E−06	1.42E−03	−1.47
*BMX*	5.20E−04	3.06E−02	−1.39
*TM4SF20*	5.73E−05	7.11E−03	−1.36
*MGAM*	8.49E−07	3.42E−04	−1.35
*ADC*	6.36E−11	8.38E−08	−1.34
*PLIN1*	3.02E−03	9.86E−02	−1.26
*CCL11*	2.98E−06	7.84E−04	−1.25
*AGBL2*	3.67E−04	2.46E−02	−1.25
*OGDHL*	1.44E−03	6.29E−02	−1.23
*RP11-1220K2.2*	2.67E−07	1.39E−04	−1.23
*SLC3A1*	6.40E−05	7.42E−03	−1.19
*HLA-DRB5*	1.79E−12	3.66E−09	−1.19
*KCNG3*	2.54E−04	1.92E−02	−1.18
*C11orf93*	2.18E−05	3.42E−03	−1.18
*LIPC*	4.44E−04	2.74E−02	−1.15
*PLEKHS1*	4.39E−04	2.74E−02	−1.15
*SCUBE2*	1.25E−04	1.19E−02	−1.15
*GRAMD2*	6.57E−04	3.61E−02	−1.14
*ROS1*	6.49E−04	3.61E−02	−1.14
*TMEM63C*	7.75E−06	1.71E−03	−1.13
*KIAA1324L*	9.14E−06	1.87E−03	−1.11
*SLC52A1*	9.49E−05	9.80E−03	−1.10
*EYA2*	1.49E−04	1.34E−02	−1.07
*BMP3*	2.09E−05	3.32E−03	−1.07
*MOCS1*	3.45E−06	8.94E−04	−1.02

### FMT suppresses bile acid secretion and olfactory transduction pathways.

To identify functional differences in gene expression due to FMT, the DEGs between pre- and post-FMT were analyzed for overrepresented pathways. The DEGs from the grouped model controlling for patient differences were used, because this model was representative of significant gene expression differences common across all patients. Gene set enrichment analysis was analyzed in the KEGG human pathway database (30). A total of 7 pathways were significantly overrepresented (FDR < 0.1) among genes suppressed after FMT (Fig. 2D). Functional overlaps in enriched pathways were identified for 6 of the 7 pathways found (Fig. 2E). The overlapping pathways were (in order of decreasing gene set size) bile secretion, metabolism of xenobiotics by cytochrome P450, chemical carcinogenesis–DNA adducts, drug metabolism–cytochrome P450, steroid hormone biosynthesis, and pentose and glucuronate interconversions. Bile secretion and cytochromes P450 are related to oxidative stress, toxin elimination, and drug processing (31, 32). Bile secretion can also be directly regulated by monooxygenases like cytochromes P450 (32). The repression of these pathways indicates a heightened transcriptional response against foreign invaders or the buildup of reactive oxygen species before FMT and a return to homeostasis after FMT. Of these overlapping pathways, the two most significant pathways were the bile secretion pathway (FDR = 0.074; enrichment score = −0.65) and the olfactory transduction pathway (FDR = 0.035; enrichment score = −0.74), which was not functionally linked to any of the other overrepresented pathways. Secondary bile acids have been shown to inhibit cell division of C. difficile (33). It was shown previously that olfactory receptors are present all along the gastrointestinal tract and can be modulated by the intestinal microbiota (34). The activation of odorant receptors has been linked to the inhibition of cell proliferation and apoptosis (35).

### Peripheral Th17 subsets are decreased following FMT.

**FIG 3:**
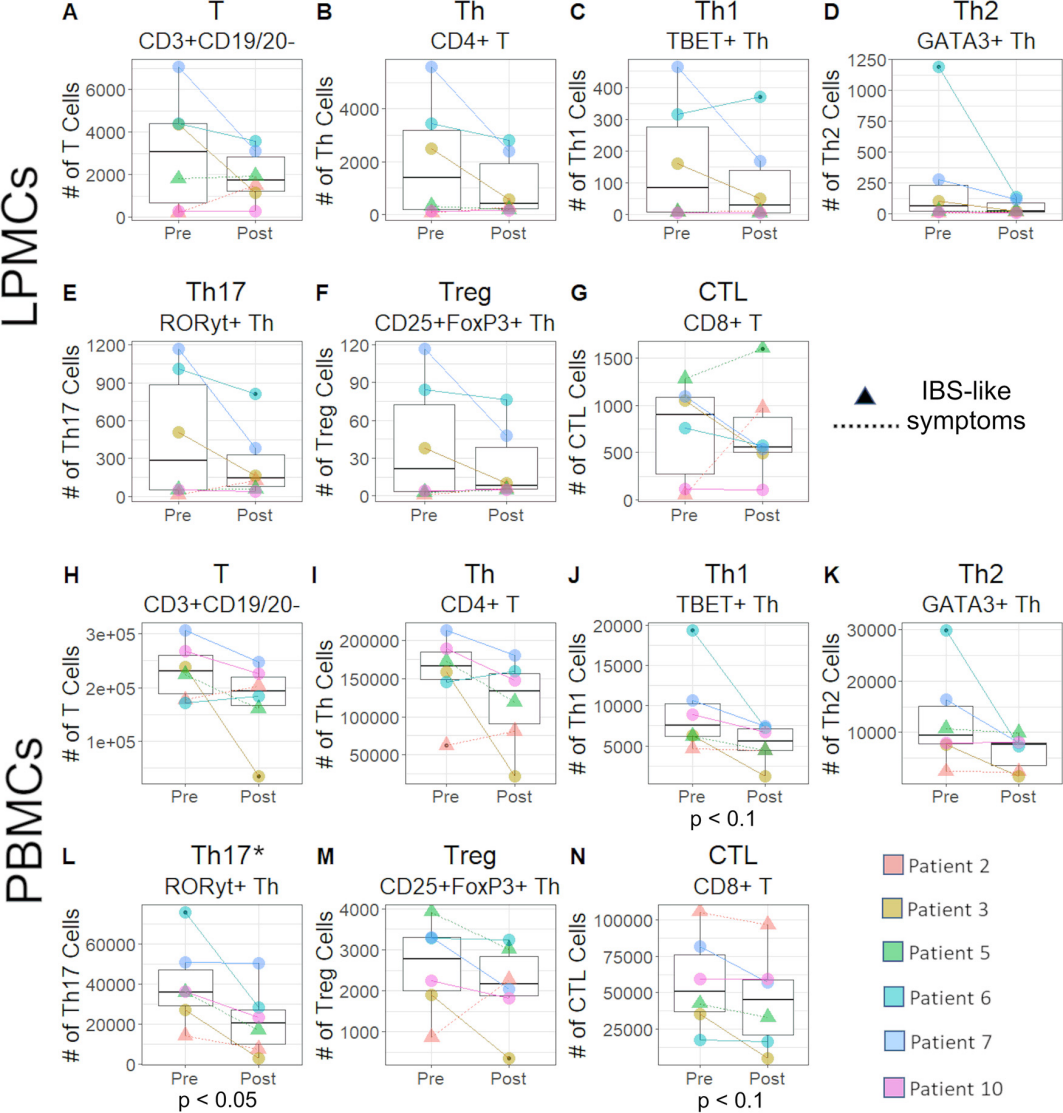
Peripheral Th17 cells were decreased after FMT (*P* < 0.05). Various T cell subsets were quantified from LPMCs (A to G) and PBMCs (H to N). A decrease in peripheral Th17 cells was observed for all patients except patient 7, who had about the same number of Th17 cells pre- and post-FMT (L). The numbers of peripheral Th1 (J) and cytotoxic T cells (CTL) (N) were decreased post-FMT; however, these decreases were not statistically significant. There were no significant differences in the number of viable CD45^+^ cells between pre- and post-FMT, nor were there significant differences in Th17 cells between patients that developed IBS-like symptoms post-FMT and those that did not (*P* > 0.1). A linear mixed effect model with patient as a random variable was used to calculate *P* values.

To complement the whole tissue RNA-seq analyses, we used high-dimensional flow cytometry to profile immune cell subsets in colonic biopsy specimens and blood from before and after FMT. Lamina propria mononuclear cells (LPMCs) were isolated from colonic biopsy specimens, and peripheral blood mononuclear cells (PBMCs) were isolated from venous blood samples. Overall, we isolated an average of 3,291 ± 2,481 (mean ± standard deviation [SD]) viable CD45^+^ cells from the LPMCs from each patient and an average of 255,397 ± 80,841 viable CD45^+^ cells from the PBMCs. *t*-distributed stochastic neighbor embedding (t-SNE) plots were used to visualize cell populations in LPMC and PBMC samples and did not reveal any immune cell subsets unique to pre- or post-FMT (Fig. S4). Statistical analyses could not be used to compare clusters between pre- and post-FMT, as specific subsets of interest appeared in various proportions overlapping one another and t-SNE does not account for differences in the relative number of cells. Therefore, we used traditional gating to test for differences in subsets of interest. This revealed a significant decrease (*P* = 0.05; linear mixed effect model with patient as a random variable) in the peripheral Th17 (CD3^+^ CD19/CD20^−^ CD4^+^ RORyt^+^) after FMT (Fig. 3L); a similar trend was noted in LPMCs, but this did not reach statistical significance (Fig. 3E). Despite this increase, there were no statistically significant differences in Th1/Th2 or Th17/Treg ratios in LPMCs or PBMCs (data not shown).

10.1128/mSphere.00669-21.4 FIG S4 t-SNE groupings did not show any immune cell populations unique to pre- or post-FMT in both LPMCs and PBMCs. The analyses were done separately between pre- and post-FMT in order to highlight any immune cell populations that were unique to pre- or post-FMT, here visualized in different colors. A contour map was used to show groupings within each immune cell subset. The color schemes and sizes of groupings were similar from pre- to post-FMT, showing no obvious differences in immune cell populations between pre- and post-FMT. Download FIG S4, TIF file, 0.9 MB. Copyright © 2021 Jan et al. 2021 Jan et al. https://creativecommons.org/licenses/by/4.0/ This content is distributed under the terms of the Creative Commons Attribution 4.0 International license.

### FMT increased microbial diversity.

**FIG 4:**
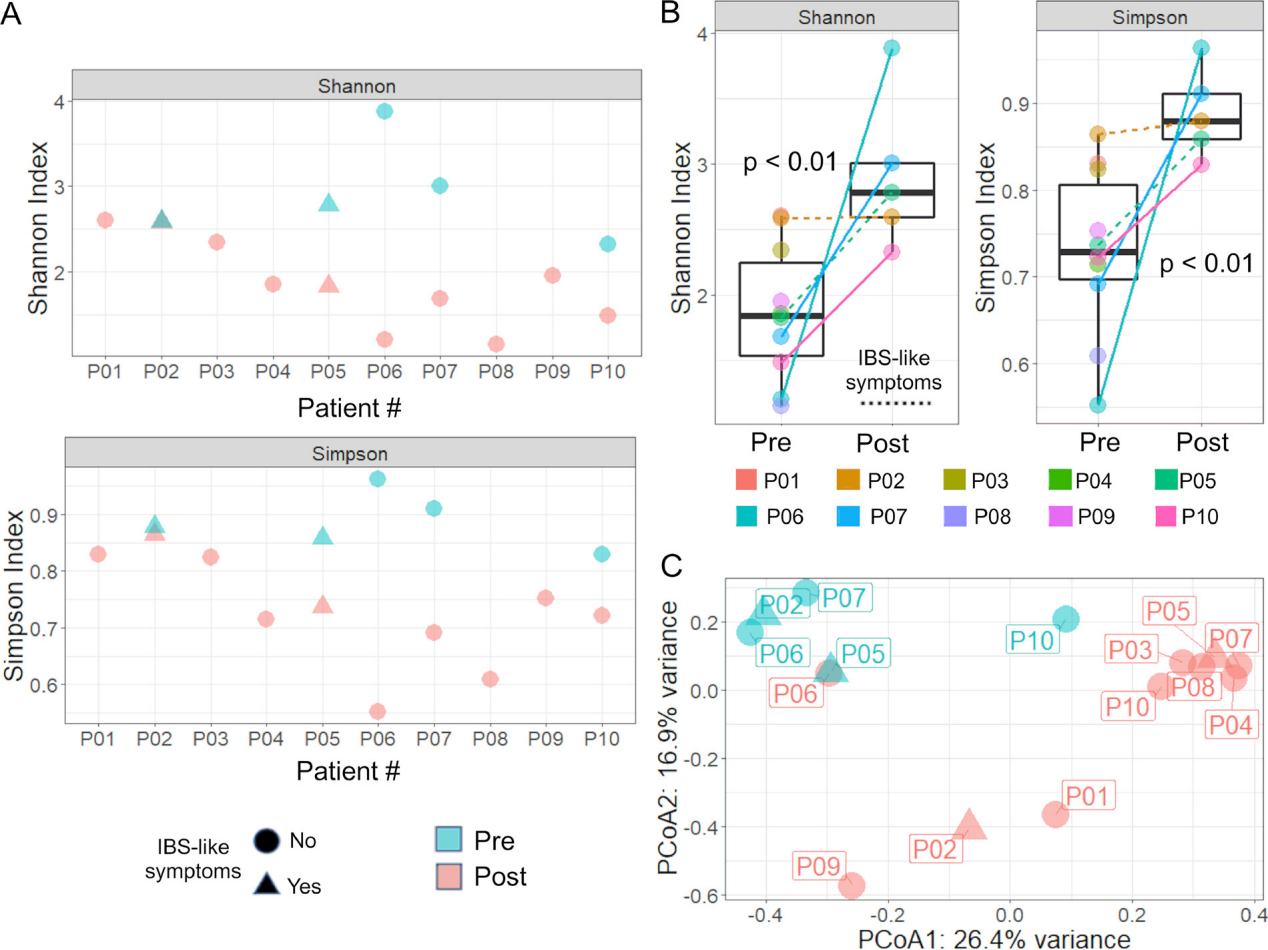
FMT increased alpha diversity of the epithelial-associated microbiome. Increased Shannon (A) and Simpson (B) alpha diversity measures were observed in 4 of the 5 patients analyzed (*P* < 0.01, Mann-Whitney test). Patient 2 (P02) showed no change in diversity post-FMT and developed IBS-like symptoms post-FMT. (C) PCoA plot showing separation of relative abundance of microbiota populations before and after FMT. This separation was statistically significant (*P* < 0.01, PERMANOVA). One point that overlapped between pre- and post-FMT was from a patient (P5) that developed IBS-like symptoms post-FMT.

To evaluate the local changes in the microbial population by FMT, 16S rRNA gene sequencing was performed using DNA isolated from biopsy samples. The use of a biopsy specimen allows us to investigate the mucosal microbial population in close association with the colonic epithelium, as opposed to the lumen of the gastrointestinal tract. We found that the alpha diversity measures Shannon and Simpson indices significantly increased after FMT (Mann-Whitney, *P* < 0.01) (Fig. 4A and B), which agreed with other studies on diversity changes after FMT treatment for rCDI (36, 37). For the beta diversity measures, clear groupings between pre- and post-FMT samples were evident in a principal-coordinate analysis (PCoA) plot (Fig. 4C). PCoA axis 1 explained 26.4% of the variance, while axis 2 explained 16.9% and separated the patients by FMT status (permutational multivariate analysis of variance [PERMANOVA], alpha = 0.01). Patients that developed post IBS-like symptoms following FMT did not appear distinct in the PCoA analysis.

The top 20 most abundant amplicon sequence variants (ASVs) across all samples were grouped by taxonomic information and ranked. In the most abundant ASVs, the top families were *Enterobacteriaceae*, *Akkermansiaceae*, *Bacteroidaceae*, *Clostridiaceae*, *Acidaminococcaceae*, *Rikenellaceae*, *Fusobacteriaceae*, *Lachnospiraceae*, *Ruminococcaceae*, and *Campylobacteraceae*, in decreasing order of abundance. The top genera from the same list of ASVs were Escherichia/*Shigella*, *Akkermansia*, *Bacteroides*, *Clostridium sensu stricto* 13, *Phascolarctobacterium*, *Alistipes*, *Lachnoclostridium*, *Clostridium sensu stricto* 1, *Agathobacter*, *Faecalibacterium*, *Fusicatenibacter*, Campylobacter, and *Cetobacterium*, in decreasing order of abundance. Some notable taxa include the genera Escherichia*/Shigella*, *Akkermansia*, and *Bacteroides*, which had different abundances between pre- and post-FMT. Escherichia*/Shigella* was found to be more abundant prior to FMT, which agrees with previous studies (38, 39). *Akkermansia* decreased after FMT, while *Bacteroides* was increased after FMT. Akkermansia muciniphila plays a role in degrading mucins in the gut and is generally thought to have a beneficial effect on the gut to reduce inflammation (40–42). *Bacteroides* has also been shown to have beneficial effects on the gut and has been shown to increase after FMT. C. difficile has also been shown to reduce *Bacteroides* growth (43). All patients were treated with vancomycin to treat C. difficile before FMT (Table 1), which may have had impacted the microbiota. A total of 15 ASVs were found to be differentially abundant between pre- and post-FMT (FDR < 0.05) (Table 3). All differentially abundant ASVs were also present as one of the top 20 most abundant ASVs. The average transcriptional fold change of genes related to bile secretion was positively correlated (*r* > 0.5) to differences in *Akkermansia* (Fig. S2E). *Akkermansia* abundance was negatively correlated (*r* < −0.7) with IL-25 protein concentration (Fig. S2F).

**TABLE 3:** Differentially abundant microbiota sorted by decreasing FDR (Wald test with Benjamini-Hochberg adjustment)

Log_2_FC	*P* value	FDR	Family	Genus	Species
−26.0	3.50E−16	1.61E−14	*Akkermansiaceae*	*Akkermansia*	*A. muciniphila*
23.2	1.21E−13	2.46E−12	*Lachnospiraceae*	*Agathobacter*	NA
23.1	1.60E−13	2.46E−12	*Ruminococcaceae*	*Faecalibacterium*	NA
−23.1	5.39E−13	6.20E−12	*Enterobacteriaceae*	Klebsiella	NA
−22.8	1.01E−12	9.33E−12	*Clostridiaceae*	*Clostridium sensu stricto 1*	*C. paraputrificum*
7.40	4.34E−03	0.0333	*Rikenellaceae*	*Alistipes*	*A. putredinis*
6.54	7.63E−03	0.0501	*Bacteroidaceae*	*Bacteroides*	B. vulgatus
7.95	0.0109	0.0570	*Bacteroidaceae*	*Bacteroides*	B. uniformis
7.80	0.0124	0.0570	*Bacteroidaceae*	*Bacteroides*	*B. caccae*
7.04	0.0113	0.0570	*Lachnospiraceae*	*Anaerostipes*	*A. hadrus*
7.25	0.0203	0.0778	*Lachnospiraceae*	*Agathobacter*	NA
7.33	0.0190	0.0778	*Bacteroidaceae*	*Bacteroides*	*B. finegoldii*
7.00	0.0251	0.0887	*Bacteroidaceae*	*Bacteroides*	NA
−5.02	0.0309	0.0947	*Clostridiaceae*	*Clostridium sensu stricto* 13	*C. subterminale*
6.77	0.0301	0.0947	*Rikenellaceae*	*Alistipes*	*A. shahii*

## DISCUSSION

The most important finding of this study was that the cytokine IL-25 was increased after FMT. This supports a model where FMT acts to protect from rCDI by restoration of commensal-bacterium signaling to the innate immune system via colonic epithelial IL-25. We demonstrated previously in the mouse model of CDI that the expression of IL-25 was rescued by FMT and that IL-25 protected from CDI via type 2 immune responses in the gut (15). The finding that IL-25 is significantly increased in rCDI patients following successful FMT validates work in mice on the importance of this cytokine in the restoration of homeostasis post-FMT. IL-25 has been shown to induce Th2-associated phenotypes like eosinophilic infiltration and increased mucus production in the gastrointestinal tract (44). IL-25 has also been shown to increase eosinophil numbers as a protective response during C. difficile infection (15).

Additional cytokines that were increased post-FMT included IL-1b and IL-4. IL-1 been shown previously to influence the role of CD4 T cells (45), and in particular, IL-1b has been shown to enhance Th2 differentiation (46). IL-1b has also been shown to be induced by C. difficile toxins in mice (17). A previous study found that IL-1b levels increased after vancomycin treatment (47), though this effect did not persist after vancomycin was discontinued. IL-4 has been shown to be important in Th2 responses, with IL-4 transcription being necessary for Th2 responses in mice (48, 49).

FMT treatment of rCDI patients also had significant effects on the epithelial transcriptional response, circulating immune cell population, cell-signaling proteins, and the mucosal microbiota. FMT induced the transcription of genes related to gut maintenance and integrity, including homeobox genes and laminins, and repressed the transcription of immune-related genes (*CCL11* and *HLA-DRB5*), detoxification (related to bile secretion and cytochromes P450 pathways), or apoptotic genes (related to olfactory transduction pathway), dampening tissue inflammation.

Th17 cells in peripheral blood were decreased post-FMT. We have previously demonstrated in the mouse model of CDI that Th17 cells exacerbated CDI in part through the production of IL-17A and recruitment of neutrophils to the colon (50). Other studies (50–52) have also seen associations between Th17 cells and CDI, where Th17 cells were found to be significantly reduced in patients with rCDI versus new-onset CDI, and found that these Th17 cells were increased after FMT. This disagrees with our finding that FMT decreases the number of Th17 immune cells, though we saw this decrease only peripherally and not locally in the colonic tissue cells. It is important to note that this previous study focused on toxin B-specific T cells as opposed to total T cells. The differences between these previous findings and our study underscore the possibility that there may be distinct differences between the immune responses of toxin-specific immune cells and the overall state of the immune system during rCDI, or an immune system in CDI remission due to vancomycin treatment.

Two general themes emerge from our results. One is that after FMT, there is a transcriptional signature of dampened inflammatory immune responses in the colon. This is evidenced by the repression of key immune response-related chemokines, the suppression of pathways related to detoxification, and the decreased olfactory signal transduction, which can be used to detect toxins or to inhibit apoptosis. This seems to be partially contradicted by the decrease in Akkermansia muciniphila, since this bacterium is generally inversely associated with inflammation (53). This may be a result of vancomycin treatment prior to FMT. Studies have shown that vancomycin can increase the abundance of Akkermansia muciniphila, which can decrease C. difficile infection (54–56).

The second theme is the progression toward healing post-FMT, as evidenced by the induction of homeobox and laminin genes related to cell proliferation as well as the increase in IL-25 tissue cytokines, which indicate a type-2 anti-inflammatory response. IL-4, another marker of a type-2 anti-inflammatory response was also found to be increased post-FMT, though this was not significant. This increase in cytokine concentration did not match an increase in the transcriptional response of eosinophil-related processes, though, so we cannot be sure if this increase is directly related to eosinophil recruitment. The increases in alpha diversity and *Bacteroides* are also a sign of healing post-FMT, as previous studies have shown that an increased microbial diversity and increased abundance of *Bacteroides* are related to the success of FMT for treating C. difficile (57, 58).

One of the limitations of our study was that we could not decouple the effects of CDI recovery from that of FMT only, as it is not feasible to recruit healthy patients with no history of CDI for the same FMT procedures. Future studies may be supplemented by animal models in order to aid in differentiating between these conditions. However, our study still analyzes data directly from clinical patients and can analyze the effects of FMT treatment for rCDI in individual patients.

Another limitation of our study was the experimental challenges associated with using colonic biopsy specimens as the source of samples. It is difficult to acquire a large amount of biomass, which can affect subsequent analyses, including LPMC isolation and DNA amplification (59, 60). For flow-cytometric analyses of the infiltrating local immune cell population, the numbers of cells isolated and analyzed from the LPMCs derived from biopsy specimens were low compared to the number of cells isolated from the blood. For one patient, the total numbers of viable CD45^+^ cells were below 500 for both pre- and post-FMT samples. To control for these differences, we analyzed immune cell phenotype differences using a mixed-effects model that accounts for patient-to-patient differences. The microbiota analysis also suffered from low recovery of bacterial DNA relative to fecal samples, but an advantage of our analysis of epithelium-associated species is a more accurate snapshot of the microbiota in close proximity to the epithelium in the specific region of the colon where FMT is expected to act (60).

In addition to the limitations mentioned above, our study was also limited to the small number of patients that completed the study. Only 6 of the 10 originally enrolled patients completed the study by coming back 60 days post-FMT. To account for this, when appropriate, unpaired patient analyses collected pre-FMT were still considered in the analyses. For example, in our 16S rRNA gene analyses of microbiome differences pre- and post-FMT, our beta diversity test on differences between pre- and post-FMT included diversity measures from patients that did not complete the study. There was a high variability in certain measures from patient to patient, including the frequency of different immune cell populations in the blood. The large variability made it difficult to interpret group trends, since there was no universal baseline for these immune cells pre-FMT. However, the inclusion of pairwise analyses made it possible to account for patient-to-patient variability. This variability was larger in transcriptomic analyses and flow-cytometric analyses than in host microbiome compositional analyses. These differences emphasize that generalizations may need to take patient-to-patient differences into account.

### Conclusions.

From our pairwise analyses, we found that rCDI patients had transcriptional evidence of dampened inflammatory responses and increased cell proliferation and healing after FMT. The exact relationships between transcriptomics, immune cell population, and microbiota may be different from person to person due to the complex nature of these interacting components of intestinal health. Our analyses show that population trends were evident in microbiota compositional analyses, but incorporating relative differences by patient increased the sensitivity of the transcriptional analysis due to higher interpatient variability in transcriptomes compared to microbiota composition. The finding that FMT increased the concentration of IL-25 indicates that anti-inflammatory eosinophil recruitment may be part of the mechanism behind FMT treatment of rCDI. The role of IL-25 in restoring mucosal homeostasis merits further investigation, as IL-25 could be a potential adjuvant to FMT for treatment of rCDI.

## MATERIALS AND METHODS

### Study design.

The study is registered at ClinicalTrials.gov (identifier NCT02797288). The goal of this study was to identify the immune mechanisms underlying successful FMT for rCDI. Specifically, we tested if FMT increased IL-25 signaling, which has been identified as initiating a protective type 2 immune response in the mouse model of CDI. Participants were recruited from patients undergoing FMT treatment for recurrent C. difficile infection as a part of their medical care at the University of Virginia Health System (UVAHS). All patients underwent standard vancomycin antibiotic treatment prior to FMT. Colonic biopsy specimens and whole blood were collected immediately prior to FMT and 60 days after FMT. A total of 10 participants were recruited (Table 1). Biopsy samples were obtained from the sigmoid colon in subjects at the time of fecal transplantation. Follow-up biopsy specimens were obtained from the sigmoid colon 60 days after FMT for six subjects. The primary outcome measure was colonic tissue IL-25 concentration. It was hypothesized that successful FMT would restore IL-25 and IL-4 in the colon. Biopsy samples taken for research purposes at each colonoscopy were analyzed to determine tissue levels of the immune signaling proteins IL-25, IL-4, and IL-1, gene expression by RNA sequencing, infiltrating and circulating immune cells using high dimensional flow-cytometry, and microbiota changes by 16S rRNA gene sequencing.

### Luminex immunoassay.

Colonic biopsy specimens were snap-frozen in liquid nitrogen and subsequently stored at −80°C until protein extraction. The biopsy specimens were lysed in MAP (multianalyte profiling) lysis buffer (Millipore) with Halt proteinase inhibitors (Thermo Fisher) using 5-mm steel beads (Qiagen) with a bead beater (TissueLyser II; Qiagen). After centrifugation, the supernatants were transferred to −80°C until use in immunoassays. The lysates were thawed, and a 47-plex magnetic bead-based Luminex immunoassay kit (Milliplex; Millipore EMD) was used to measure protein concentrations. The mean fluorescence intensity (MFI) for each protein was read using Luminex MagPix and analyzed using Milliplex Analyst 5.1 (Millipore EMD), which uses a 5-log standard fit to calculate protein concentration from MFI. For MFIs outside the range of the standards, the concentrations were extrapolated using their respective standard curves (derived from the Milliplex Analyst, back calculated using Matlab [MathWorks]). Differences in protein concentrations between pre- and post-FMT were tested for significance (alpha = 0.05) using a linear mixed effect model, where patient ID was included as a random variable (Table S2). IL-1a, IL-1b, IL-4, and IL-25 were defined *a priori* in the analysis plan, so the *P* values were not corrected for multiple comparisons. A Benjamini-Hochberg adjustment was used for the *P* values calculated for the 43 remaining proteins, since they were not preselected.

10.1128/mSphere.00669-21.6 TABLE S2 Flow cytometry panel. Download Table S2, DOC file, 0.04 MB. Copyright © 2021 Jan et al. 2021 Jan et al. https://creativecommons.org/licenses/by/4.0/ This content is distributed under the terms of the Creative Commons Attribution 4.0 International license.

### mRNA sequencing and analysis.

Colonic biopsy specimens were obtained during colonoscopy and immediately placed in Allprotect (Qiagen) and stored at −80°C until nucleic acid extraction (Qiagen AllPrep). A total amount of 1 μg RNA per sample was used as input material for the RNA sample preparations. Sequencing libraries were generated using NEBNext Ultra RNA library preparation kit for Illumina (New England Biolabs [NEB], USA) following the manufacturer’s recommendations. Briefly, mRNA was purified from total RNA using poly(T) oligonucleotide-attached magnetic beads. Fragmentation was carried out using divalent cations under elevated temperature in NEBNext first-strand synthesis reaction buffer (5×). First-strand cDNA was synthesized using random hexamer primer and Moloney murine leukemia virus reverse transcriptase, RNase H minus (M-MuLV [H^−^]). Second-strand cDNA synthesis was subsequently performed using DNA polymerase I and RNase H. Remaining overhangs were converted into blunt ends via exonuclease/polymerase activities.

After adenylation of 3′ ends of DNA fragments, a NEBNext adapter with a hairpin loop structure was ligated to prepare for hybridization. In order to select cDNA fragments of preferentially 150 to 200 bp, the library fragments were purified with AMPure XP system (Beckman Coulter, Beverly, MA, USA). Then, 3 μl USER enzyme (NEB, USA) was used with size-selected, adapter-ligated cDNA at 37°C for 15 min followed by 5 min at 95°C before PCR. Then, PCR was performed with Phusion high-fidelity DNA polymerase, universal PCR primers and index (X) primer using the NEBNext multiplex oligonucleotides for Illumina. PCR products were purified (AMPure XP system), and library quality was assessed on the Agilent Bioanalyzer 2100 system. Clustering of index-coded samples was performed on a cBot cluster generation system using the PE (paired-end) cluster kit cBot-HS (Illumina) according to the manufacturer’s instructions.

After cluster generation, the library preparations were sequenced on a MiSeq (Illumina) platform, and 150-bp paired-end reads were generated. A total of 13 samples were processed, 1 from pre-FMT and 1 from post-FMT for each of 6 patients except patient 3, who had 2 samples from pre-FMT as a control for batch effects. Raw reads were processed through fastp (61) to remove adapter sequences, poly-N sequences, and reads with low quality. Q20, Q30, and GC content of the clean data were calculated, and only high-quality reads were preserved. Paired-end clean reads were aligned to the reference genome Homo sapiens (GRCh37/hg19) using the Spliced Transcripts Alignment to a Reference (STAR) software (62). FeatureCounts was used to quantify reads mapped to each gene (63). Read counts were processed using the bioconductor package DESeq2 v1.30.1 in R (version 4.0.5) and normalized using the DESeq algorithm. Principal-component analysis (PCA), hierarchical clustering, and density maps using pheatmap v1.0.12 (64) assessed overall similarity between samples and were used to determine the importance and influence of various factors, including the effects of FMT, patient, and persistent IBS symptoms following FMT. From the PCA, top positive and negative loadings for components 1 and 2 were reported. For the density maps, a heat map was constructed from the 30 most variable genes. For each gene, the standard deviation of counts across all samples was calculated as a measure of variability. Differentially expressed genes were calculated using the Wald test in DESeq2 (65). We used two models, a grouped model and a patient-specific model. The grouped model contrasted between pre- and post-FMT while controlling for patient effects by adding patient ID as a separate variable to the model (design = ∼Patient+Visit) (*n* = 13). The patient-specific model contrasted pre- and post-FMT for each paired patient (6 total) by using a term combining patient ID and whether the sample was pre- or post-FMT (design = ∼Patient_Visit). Genes with log_2_ fold changes (log_2_FC) of ≥1 and using the Benjamini-Hochberg false discovery rate correction (FDR, 0.1) were considered significant.

### Gene set enrichment analysis.

Gene set enrichment analysis was performed on a list of all genes and their respective log_2_FCs in the grouped model. The package fgsea v3.13 (66) in R was used to analyze the fold changes of the genes to identify enriched gene pathways and functions using KEGG pathways. *P* values were calculated using an enrichment score statistic (67). Enrichment maps were constructed to determine functional modules using the emapplot function within the enrichplot v1.10.1 package in R. The resulting enriched pathways and their corresponding gene lists were then selectively narrowed for each patient using the patient-specific model to measure the extent of enrichment for a pathway for each patient. As a measure of overall FC, the log FCs for the differentially expressed genes were summed for each enriched gene pathway of interest for each patient with paired RNA-seq data between pre- and post-FMT. This sum represented the extent of enrichment for a given pathway for that patient.

### Flow cytometry of LPMCs and PBMCs.

Lamina propria mononuclear cells (LPMCs) were isolated from colonic biopsy specimens as follows. Colonic biopsy specimens were submerged in ice-cold Hanks balanced salt solution (HBSS) without calcium and magnesium and immediately transported to the laboratory for cell disassociation. The biopsy specimens were digested at room temperature for 20 min (Accumax; eBioscience) and filtered (70 μm) to isolate LPMCs. For PBMC isolation, whole blood was centrifuged and the plasma removed. The PBMCs were isolated by adding Ficoll (Cytiva) and then centrifuged in SepMate tubes (Stemcell Technologies). Isolated LPMCs and PBMCs were cryopreserved in liquid nitrogen until staining.

Isolated LPMCs and PBMCs were thawed and stained with fluorochrome-conjugated antibodies (Table S1). Unstained, single-stained, and fluorescence-minus-one (FMO)-stained PBMCs were used as controls. In addition, each sample also underwent a fixed viability stain (Zombie NIR; BioLegend). Cells were first stained with surface stains and then permeabilized (Foxp3 transcription factor staining buffer set; Invitrogen) for intracellular staining. Samples were analyzed with the five-laser Cytek Aurora Borealis flow cytometer. All cells were collected for LPMC samples and 100,000 cells for PBMC samples. These fluorescence reads were then analyzed using FlowJo V.10.7.2 to phenotype immune cell subsets via t-SNE clustering and traditional gating (Fig. S3). Spectral deconvolution and gating were based on single-stained and FMO-stained PBMC control samples.

For each t-SNE cluster or immune-cell phenotype of any given sample, the raw counts and percentage of leukocyte population (viable CD45^+^ cells) were calculated. Differences in these raw counts and percentages between pre- and post-FMT were quantified using a linear mixed effect model, where patient ID was included as a random variable.

### 16S rRNA gene sequencing and analysis of epithelial-associated microbiota.

Bacterial DNA from colonic biopsy specimens was amplified using V4 specific primers and indexed using Nextera XT index kit, which was then sequenced by Illumina via MiSeq sequencing (68). The amplification was insufficient from one post-FMT sample, narrowing the number of samples analyzed (10 pre-FMT and 5 post-FMT samples). A mock sample (ZymoBIOMICS) was included as a control. The resulting library was preprocessed through the DADA2 package (69) in R to form a library of amplicon sequence variants (ASVs). Taxonomy was assigned with Silva (70) release 138 databases. Forward reads were truncated at 240 bp, while reverse reads were truncated at 160 bp. A paired-end library was used with an expected amplicon size of around 250 bp. This length was confirmed after merging forward and reverse reads in the DADA2 pipeline.

The alpha diversity measures Shannon and Simpson diversity values were calculated using the phyloseq (71) v.1.34.0 library in R. Differences in alpha diversity measures were assessed between pre- and post-FMT using the pairwise.wilcox.test base function in R with a Bonferroni correction for multiple comparisons. This function uses a paired, ranked Mann-Whitney test to evaluate differences.

Principal-coordinate analysis (PCoA) was performed to visualize the differences in relative abundance of pre- and post-FMT microbiota. Differences between pre- and post-FMT were evaluated by permutational multivariate analysis of variance (PERMANOVA) in the vegan package (72) v.2.5.7 of R.
