# Supplementary material for: Fecal Microbiota Transplantation Increases Colonic IL-25 and Dampens Tissue Inflammation in Patients with Recurrent Clostridioides difficile
Source: mSphere. 2021 Oct 27;6(5):e00669-21. doi: 10.1128/mSphere.00669-21 (PMC8550158; doi:10.1128/mSphere.00669-21)
Supplement: TABLE S1 [file msphere.00669-21-s0005.doc]

| **Table S1. Pre vs Post FMT protein concentrations from Luminex assay (linear mixed controlled by patient with Benjamini-Hochberg adjustment).** | | | | | | | |
| --- | --- | --- | --- | --- | --- | --- | --- |
|  |  | Pre FMT | | Post FMT | |  |  |
| Type | Analyte | Median (pg/mL) | IQR (pg/mL) | Median (pg/mL) | IQR (pg/mL) | *p* value | FDR |
| Chemokine | MCP-1 (CCL2) | 110.0 | 32.5 | 147.6 | 116.7 | 0.353 | 0.405 |
|  | MIP-1α (CCL3) | 17.7 | 18.6 | 31.1 | 11.3 | 0.235 | 0.307 |
|  | MIP-1β (CCL4) | 39.6 | 9.3 | 39.9 | 20.6 | 0.824 | 0.824 |
|  | MCP-3 (CCL7) | 13.9 | 4.4 | 35.7 | 11.0 | 0.00950 | 0.102 |
|  | EOTAXIN-1 (CCL11) | 25.3 | 15.2 | 27.0 | 7.9 | 0.455 | 0.497 |
|  | MDC (CCL22) | 28.6 | 14.0 | 24.0 | 21.4 | 0.710 | 0.726 |
|  | GROα (CXCL1) | 86.5 | 66.2 | 267.6 | 132.1 | 0.136 | 0.208 |
|  | IL-8 (CXCL8) | 23.1 | 24.9 | 36.0 | 25.4 | 0.265 | 0.328 |
|  | MIG (CXCL9) | 23799.5 | 17844.5 | 4073.0 | 8637.3 | 0.0183 | 0.102 |
|  | IP-10 (CXCL10) | 379.0 | 432.9 | 62.9 | 95.6 | 0.147 | 0.209 |
|  | Fractalkine (CX3CL1) | 72.9 | 11.8 | 169.4 | 56.0 | 0.0232 | 0.102 |
| Cytokine | Flt-3 ligand | 11.4 | 4.1 | 12.4 | 8.1 | 0.347 | 0.405 |
|  | IFNα2 | 33.1 | 17.1 | 73.6 | 28.8 | 0.0161 | 0.102 |
|  | IFNγ | 45.9 | 18.8 | 155.7 | 105.7 | 0.0347 | 0.102 |
|  | **IL-1α** | 11.8 | 3.8 | 26.0 | 8.6 | 0.0424 | 0.102 |
|  | **IL-1β** | 9.7 | 10.3 | 28.8 | 10.8 | 0.0315 | 0.102 |
|  | IL-1RA | 1080.5 | 969.4 | 3475.5 | 1246.3 | 0.0218 | 0.102 |
|  | IL-2 | 0.2 | 0.1 | 0.9 | 0.6 | 0.151 | 0.209 |
|  | IL-3 | 2.1 | 13.3 | 37.5 | 4.5 | 0.0044 | 0.0689 |
|  | **IL-4** | 10.0 | 3.3 | 20.0 | 5.0 | 0.0583 | 0.125 |
|  | IL-5 | 0.8 | 0.2 | 2.2 | 1.1 | 0.0307 | 0.102 |
|  | IL-6 | 1.6 | 0.7 | 2.9 | 1.6 | 0.147 | 0.209 |
|  | IL-7 | 3.1 | 11.2 | 11.8 | 18.7 | 0.277 | 0.333 |
|  | IL-9 | 30.1 | 7.9 | 91.7 | 30.6 | 0.0040 | 0.069 |
|  | IL-10 | 149.2 | 87.9 | 441.4 | 242.1 | 0.0194 | 0.102 |
|  | IL-12p40 | 15.4 | 19.4 | 21.1 | 9.1 | 0.642 | 0.670 |
|  | IL-12p70 | 1.7 | 0.2 | 4.5 | 3.2 | 0.0214 | 0.102 |
|  | IL-13 | 11.7 | 3.7 | 35.5 | 20.5 | 0.0257 | 0.102 |
|  | IL-15 | 94.3 | 10.1 | 103.8 | 59.2 | 0.1077 | 0.181 |
|  | IL-17A | 6.4 | 3.6 | 15.1 | 12.6 | 0.0400 | 0.102 |
|  | **IL-17E (IL-25)** | 13.1 | 11.1 | 41.8 | 10.1 | 0.0016 | 0.069 |
|  | IL-17F | 2.4 | 1.9 | 9.0 | 6.2 | 0.0324 | 0.102 |
|  | IL-18 | 18.6 | 14.7 | 11.1 | 2.2 | 0.245 | 0.311 |
|  | IL-22 | 142.1 | 188.0 | 317.9 | 80.4 | 0.0711 | 0.135 |
|  | IL-27 | 849.1 | 1083.4 | 1514.0 | 430.5 | 0.635 | 0.670 |
|  | TGF-α | 13.8 | 9.0 | 23.0 | 14.2 | 0.0746 | 0.135 |
|  | TNF-α | 17.6 | 8.7 | 29.1 | 24.4 | 0.374 | 0.419 |
|  | TNF-β | 4.1 | 1.4 | 15.0 | 11.0 | 0.0494 | 0.111 |
|  | sCD40L | 4105.5 | 1472.5 | 6123.0 | 3395.8 | 0.0742 | 0.135 |
| Growth factor | EGF | 25.0 | 3.9 | 51.1 | 20.3 | 0.0344 | 0.102 |
|  | FGF-2 | 9885.5 | 1934.5 | 13651.0 | 3927.5 | 0.0435 | 0.102 |
|  | G-CSF | 76.0 | 92.9 | 157.1 | 343.3 | 0.137 | 0.208 |
|  | GM-CSF | 0.0 | 0.2 | 1.6 | 1.6 | 0.0891 | 0.155 |
|  | M-CSF | 476.8 | 80.1 | 683.8 | 926.6 | 0.120 | 0.194 |
|  | PDGF-AA | 246.5 | 94.5 | 439.2 | 195.2 | 0.0652 | 0.133 |
|  | PDGF-AB/BB | 0.0 | 0.0 | 123.0 | 167.5 | 0.0376 | 0.102 |
|  | VEGF | 126.4 | 103.9 | 206.8 | 189.0 | 0.164 | 0.220 |
