# Supplementary figures and images for: Fecal Microbiota Transplantation Increases Colonic IL-25 and Dampens Tissue Inflammation in Patients with Recurrent Clostridioides difficile
Source: mSphere. 2021 Oct 27;6(5):e00669-21. doi: 10.1128/mSphere.00669-21 (PMC8550158; doi:10.1128/mSphere.00669-21)

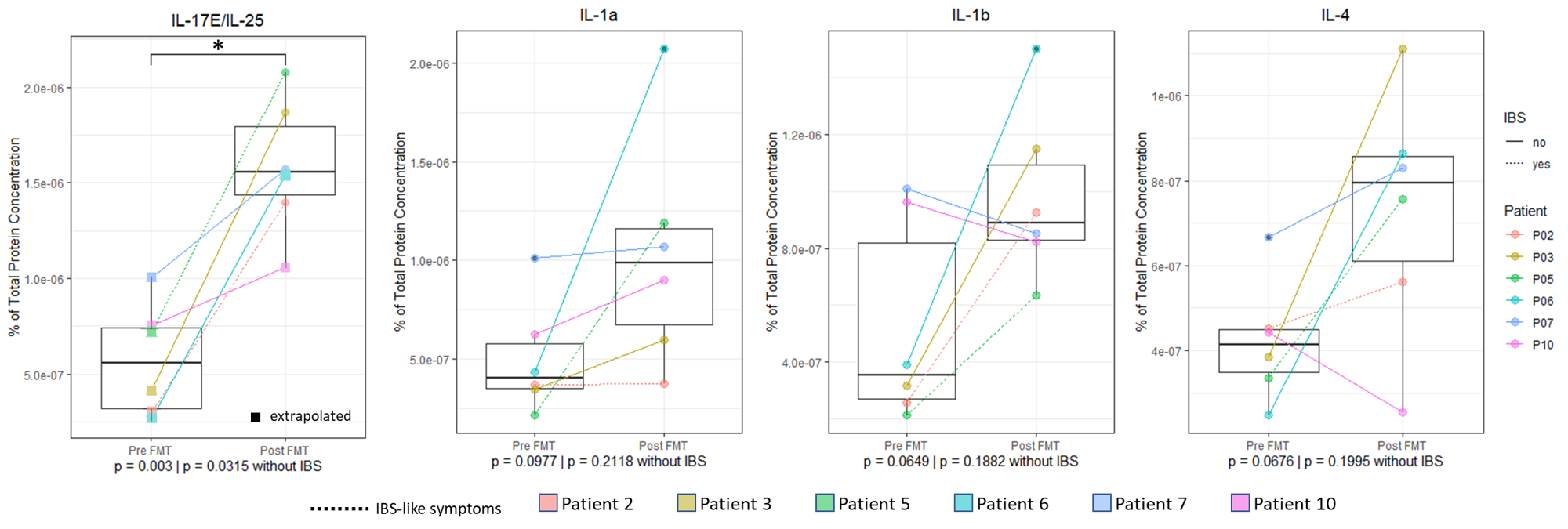

Supplement: FIG S1 [file msphere.00669-21-s0001.tif]

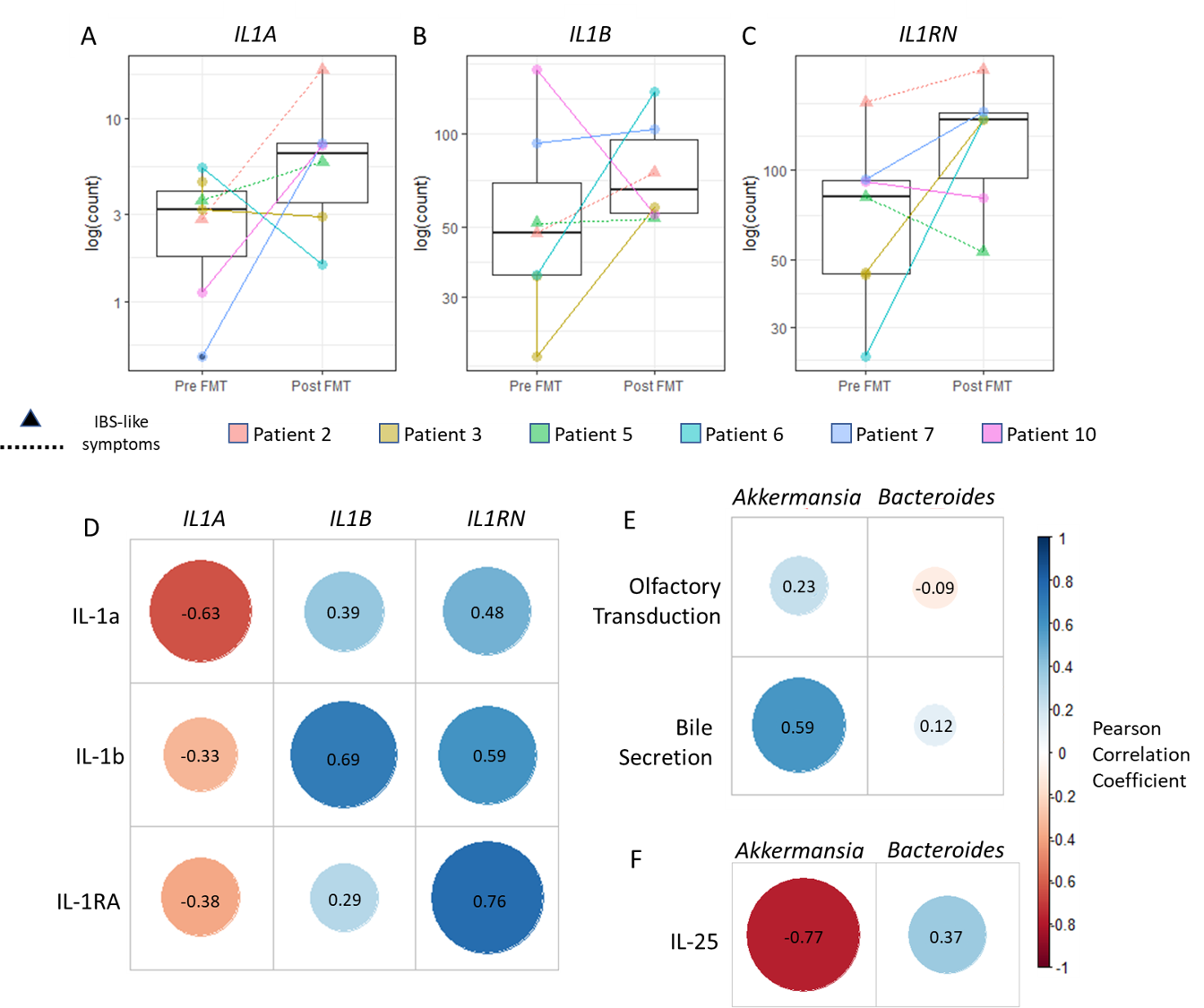

Supplement: FIG S2 [file msphere.00669-21-s0002.tif]

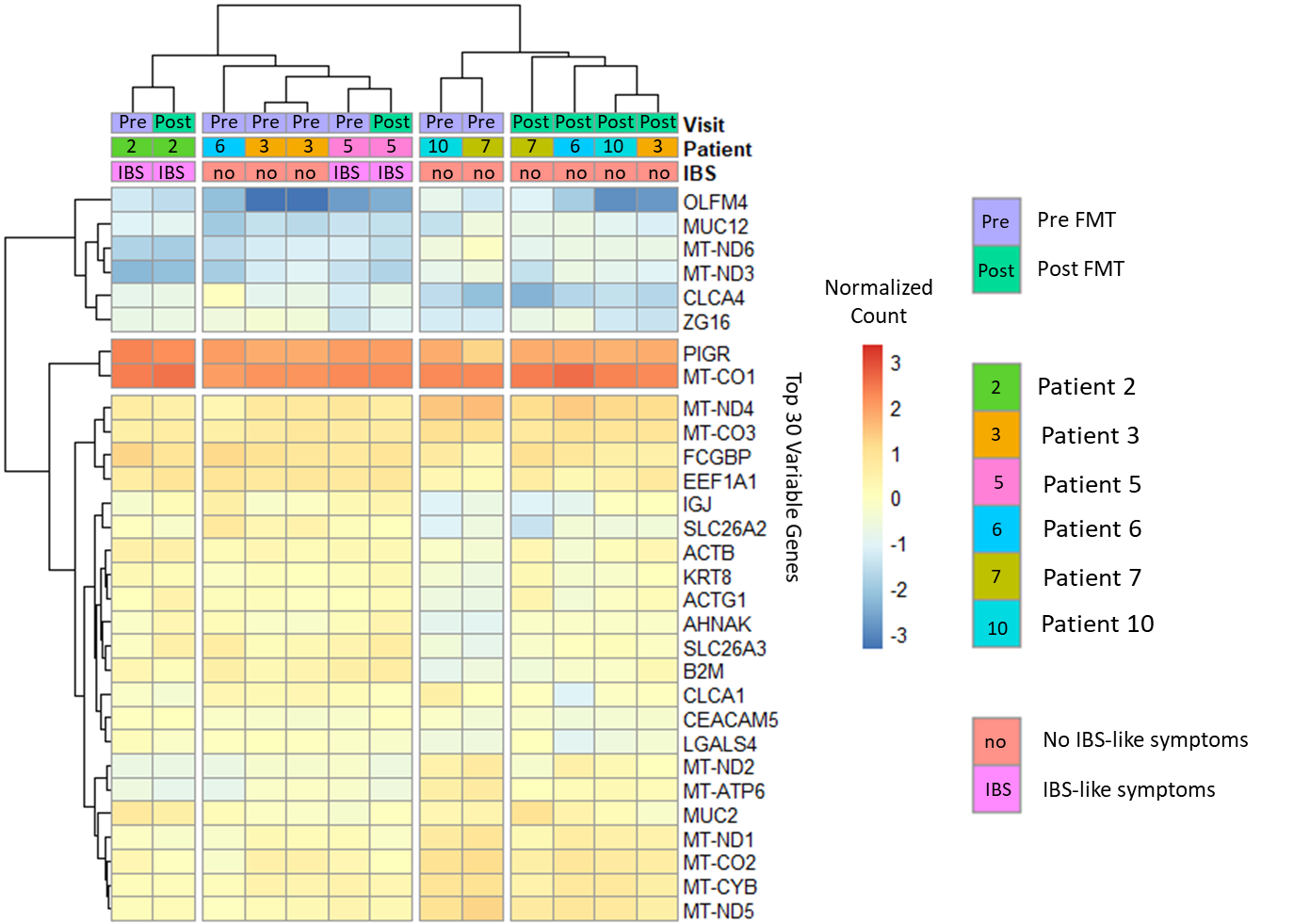

Supplement: FIG S3 [file msphere.00669-21-s0003.tif]

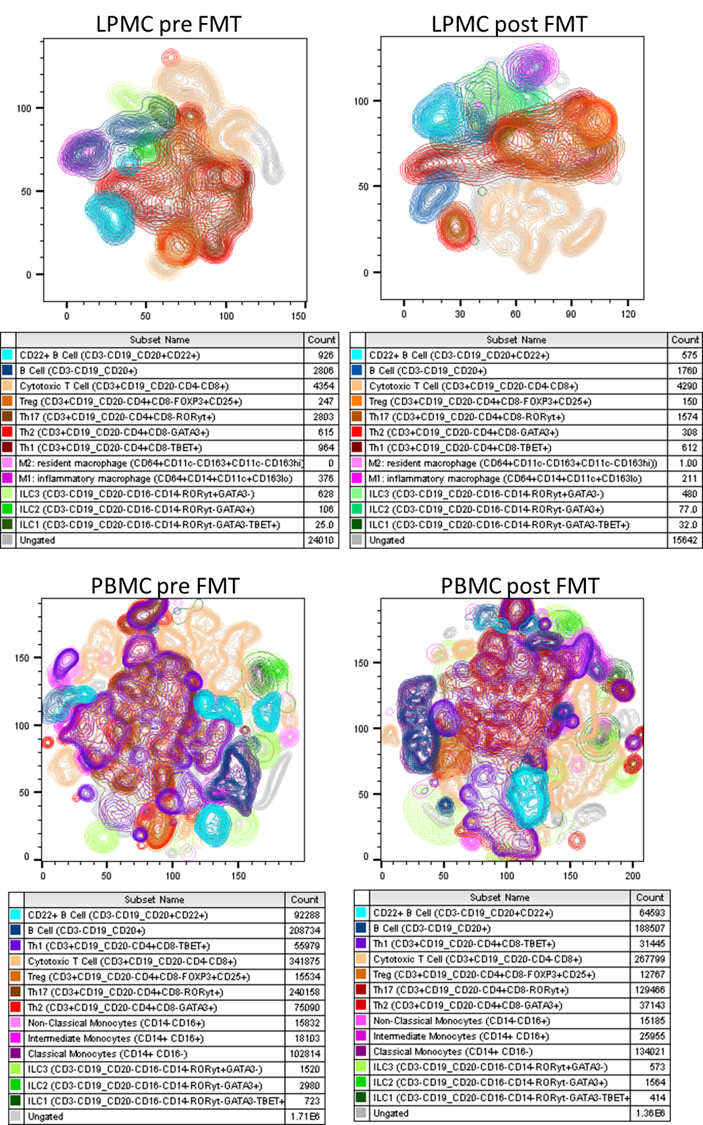

Supplement: FIG S4 [file msphere.00669-21-s0004.tif]
