# Supplementary material for: Fecal Microbiota Transplantation Increases Colonic IL-25 and Dampens Tissue Inflammation in Patients with Recurrent Clostridioides difficile
Source: mSphere. 2021 Oct 27;6(5):e00669-21. doi: 10.1128/mSphere.00669-21 (PMC8550158; doi:10.1128/mSphere.00669-21)
Supplement: TABLE S2 [file msphere.00669-21-s0006.doc]

| **Table S2. Flow Cytometry Panel** | | |
| --- | --- | --- |
| Surface Stains | Marker | Fluorophore |
|  | CCR7 | BUV395 |
|  | CD103 | BUV 563 |
|  | CD83 | BUV 737 |
|  | CD22 | BUV805 |
|  | CD14 | Pacific Blue |
|  | CD127 | eFluor506 |
|  | CD11b | BV510 |
|  | CD45RA | BV570 |
|  | CD163 | BV605 |
|  | CD4 | BV650 |
|  | CD8 | BV750 |
|  | HLA-DR | BV785 |
|  | CD45 | QDOT 800 |
|  | CD11c | BB515 |
|  | CD3 | Spark 550 |
|  | CD117 | PerCP-Cy5.5 |
|  | gdTCR | PerCP-ef710 |
|  | CD25 | PE-Dazzle 594 |
|  | CD294 | PE-Cy5 |
|  | CD16 | PE-Cy7 |
|  | CD19/20 | APC-Cy5.5 |
|  | CD56 | AF700 |
|  | CD64 | APC-fire 750 |
|  |  |  |
| Intracellular Stains | Marker | Fluorophore |
|  | FoxP3 | BV421 |
|  | Tbet | BV711 |
|  | Ki67 | AF532 |
|  | Gata3 | PE |
|  | RORyt | AF647 |
